# Microbial-derived metabolites in cancer: evidence boundaries, immune mechanisms and translational pathways

**DOI:** 10.3389/fimmu.2026.1947330

**Published:** 2026-10-12

**Authors:** Xuefeng Shi, Shunjun Wang, Wenhao Ouyang

**Affiliations:** 1 Respiratory and Critical Care Medicine Department, Qinghai Provincial People’s Hospital, Xining, China; 2 Department of Cardiology Surgery, Xiangya Hospital of Central South University, Changsha, China; 3 Guangdong Provincial Key Laboratory of Malignant Tumor Epigenetics and Gene Regulation, Department of Medical Oncology, Sun Yat-sen Memorial Hospital, Sun Yat-sen University, Guangzhou, China

**Keywords:** chemotherapy resistance, engineered bacteria, epigenetics, immunotherapy, innate immunity, microbial metabolites, oncometabolites, postbiotics

## Abstract

Microbial metabolites can suppress or promote tumor development and modify treatment response, but the direction of an effect depends on its chemical identity, anatomical source, concentration and host target. This narrative review critically examines short-chain fatty acids, tryptophan derivatives, secondary bile acids, colibactin, cyclic dinucleotides and microbial activities that alter anticancer drugs. We distinguish individual metabolites from postbiotic preparations, microbial components and enzymes, and separate microbial production from host–microbial co-metabolism. Mechanistic examples include butyrate-dependent chromatin regulation, macrophage aryl hydrocarbon receptor signaling, microbial agonists of STING, colibactin-induced DNA cross-links and bacterial conversion of gemcitabine or irinotecan metabolites. For each, we consider the experimental model, evidence for causality, physiological exposure and limits of human translation. Particular attention is given to apparently conflicting short-chain-fatty-acid findings across checkpoint inhibitors, the conditional effects of dietary fiber, and discordance between experimental drug inactivation and human pancreatic tumor profiling. Human studies support selected biomarker associations and early intervention feasibility, including fecal microbiota transplantation and engineered bacterial delivery, but do not establish a general metabolite-based treatment strategy. We therefore propose a seven-step research framework linking patient sampling, direct quantification and source attribution, causal and functional testing, external biomarker validation, intervention selection, pharmacodynamic monitoring and prospectively defined clinical endpoints. Its contribution is to make the evidence required for each translational decision explicit while distinguishing clinically useful prediction from a causally justified intervention.

## 1 Introduction

Microbial metabolism provides one route through which the microbiome can influence cancer biology and the response to treatment. The relevant processes extend beyond microorganisms physically located in a tumor: gut products may act locally on intestinal immune cells, enter the circulation, undergo host transformation or alter a drug before it reaches its target. Contemporary reviews describe this relationship across immunotherapy and drug disposition, while analysis of human tumor specimens has established that bacterial signals can also occur within cancer and immune cells (1–3). These observations motivate functional investigation, but they answer different questions. Detecting a bacterium establishes neither production of a particular compound nor an exposure sufficient to change tumor behavior. Conversely, detecting a compound in a tumor does not identify the organism or tissue that produced it. A mechanistically useful account must connect the source, molecule, host target and outcome without treating one measurement as proof of all four.

The ISAPP consensus defines a postbiotic as a preparation of inanimate microorganisms and/or their components that confers a health benefit on the host (4). A purified short-chain fatty acid (SCFA), indole or vitamin is therefore not automatically a postbiotic. We use microbial-derived metabolite for a chemically defined product of microbial metabolism and host–microbial co-metabolite where both partners contribute to its formation. Exopolysaccharides are discussed as microbial macromolecular products, whereas drug-metabolizing enzymes are catalytic proteins; neither category should be conflated with small-molecule metabolites. Colibactin is designated a microbial genotoxin because its DNA-damaging activity is directly demonstrated (5).

Gut-derived SCFAs encounter intestinal epithelial and immune cells before absorption and substantial host metabolism. Secondary bile acids originate through microbial modification of liver-derived primary bile acids, while some tryptophan products can reflect both microbial and host pathways. Intratumoral bacteria potentially generate local products, but this possibility requires direct testing. Han et al. developed a culture-based metabolomics pipeline for assigning compounds to microbial producers, providing a practical complement to sequencing (6). In pancreatic cancer, Riquelme et al. connected tumor microbial composition, immune features and outcomes and used transfer experiments to test community effects (7). Such experiments strengthen community-level biological inference without identifying every responsible metabolite. Accordingly, gut contents, feces, plasma and tumor tissue are treated as distinct sampling compartments throughout this review.

Several relevant reviews already cover much of the biological territory. Perl et al. focus on metabolite regulation of cancer immunotherapies, including efficacy and immune toxicity (1); Malczewski et al. emphasize the metabolome as a potential predictor of immunotherapy response (8). Zhang et al. examine the origins, analysis and treatment implications of the intratumoral microbiome (9), and Zalatan et al. discuss bacterial engineering, payload delivery and containment (10). The present review does not claim that the move toward functional microbiome research is itself new. Its specific contribution is a comparison across these areas using the same questions: is the compound measured directly, is its source established, does perturbation support causality, and what evidence would justify biomarker use or intervention? The unresolved gap is the frequent disconnect between a molecular mechanism, a clinical association and the decision to manipulate that mechanism therapeutically.

This is a narrative review with a targeted, question-driven literature update, not a systematic review or a meta-analysis. The revision used PubMed-indexed records retrieved through Europe PMC, publisher articles and DOI metadata to identify and check relevant literature up to 8 September 2026. Searches combined microbial or microbiota-derived metabolites with cancer, short-chain fatty acids, tryptophan, bile acids, colibactin, STING, gemcitabine, irinotecan, immunotherapy, biomarkers and engineered bacteria; exact-title and DOI searches were also used to verify studies already cited. Primary studies were prioritized when they directly measured metabolites, manipulated a producer or host pathway, tested treatment outcomes, or reported human cohorts. Non-cancer studies were included only where they clarify a mechanism or delivery limitation and are identified as such. Duplicate preprints were replaced by the corresponding peer-reviewed article when available; tangential examples and unsupported source assignments were excluded. The selection is purposive and cannot support claims of exhaustive coverage.

Evidence is appraised along two complementary dimensions: causal resolution and clinical applicability. Biochemical and cell experiments can identify a molecular reaction but usually lack an intact host; controlled animal studies can establish model-specific causality but remain sensitive to microbial ecology, immunity and exposure. Human observational studies assess relevance in patients but are vulnerable to confounding and reverse causation. Prospective biomarker cohorts improve temporal ordering, while early interventional trials can establish feasibility or target engagement without proving efficacy. Clinical validation additionally requires reproducibility and evidence that using the marker or treatment improves a relevant decision or outcome. These distinctions are consistent with the need for transparent biomarker reporting (11). **Figure 1** provides an orientation to these relationships, rather than a ranking of universally beneficial or harmful microbial products.

**Figure 1:**
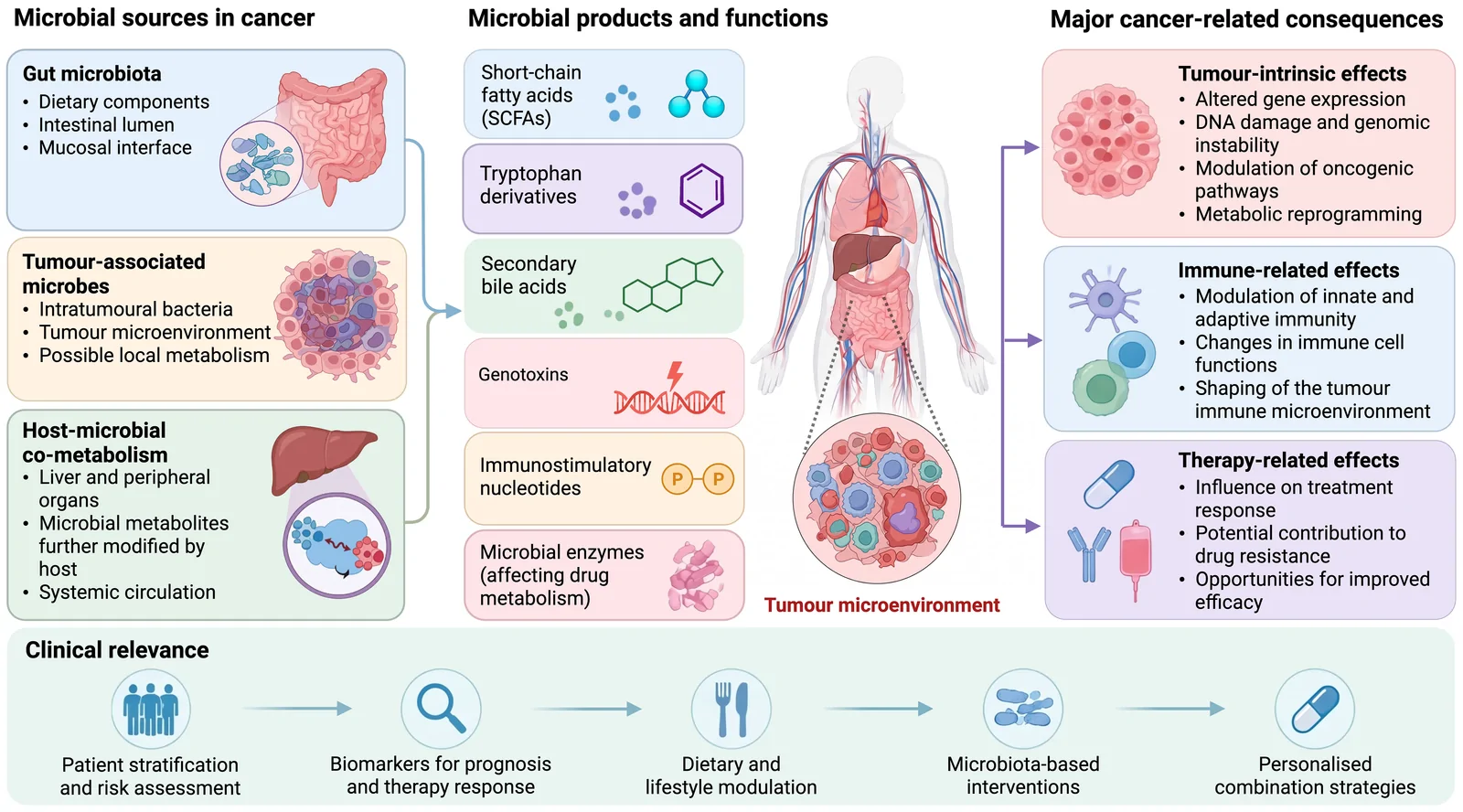
Microbial sources, products and context-dependent consequences in cancer. Gut microbiota, tumour-associated microorganisms and host–microbial co-metabolism contribute to the production, transformation and distribution of microbial products across intestinal, systemic and tumour-associated compartments. Representative products and activities include short-chain fatty acids (SCFAs), tryptophan derivatives, secondary bile acids, microbial genotoxins, immunostimulatory cyclic dinucleotides and microbial enzymes involved in drug metabolism. These products and activities may influence cancer through three broad categories of effects: (i) tumour-intrinsic effects, including changes in gene expression, DNA damage and metabolic regulation; (ii) immune-related effects, including modulation of innate and adaptive immune responses and the tumour immune microenvironment; and (iii) therapy-related effects, including altered drug activity, treatment resistance and potential therapeutic sensitisation. The direction and magnitude of these effects depend on molecular identity, exposure compartment, concentration, responding cell type and treatment context. The lower panel illustrates potential translational applications, including patient stratification, candidate biomarkers, dietary modulation, microbiota-based interventions and personalised treatment strategies. These applications represent research and development opportunities rather than uniformly validated clinical practices. Arrows indicate conceptual relationships and do not imply equivalent causal evidence or clinical validation across pathways.

### 1.1 Microbial products and host immune regulation

#### 1.1.1 Short-chain fatty acids: chromatin regulation, receptors and exposure

SCFAs are fermentation products whose physiological effects cannot be inferred solely from the label “beneficial metabolite”. Butyrate illustrates the dependence on host metabolic state. In cultured colonocytes, Donohoe et al. showed that oxidative metabolism of butyrate supplies acetyl-CoA and supports histone acetyltransferase activity, whereas glycolytic cancer cells accumulate butyrate and exhibit histone deacetylase (HDAC) inhibition (12). The two routes can both increase histone acetylation while producing different transcriptional and proliferative responses. Thus, increased histone acetylation is not by itself evidence of tumor suppression. The balance depends on substrate utilization, intracellular accumulation and the genes affected. This explains why a mechanism observed in one colorectal cell model should not be extrapolated automatically to breast, oral or other cancers with different metabolic programs.

A more direct test of the diet–microbe–metabolite relationship was provided by gnotobiotic colorectal tumor experiments using butyrate-producing bacteria and a production-defective comparator. In that setting, dietary fiber reduced tumor formation in a microbiota- and butyrate-dependent manner, with altered histone acetylation and tumor-cell proliferation (13). The producer perturbation is a major strength because it moves beyond a correlation between fiber intake and fecal composition. Nevertheless, the result depends on the defined microbial community and host model, and does not show that every fermentable fiber or butyrate-producing strain is protective. The distinction between a substrate, a producing organism and the resulting local metabolite concentration is therefore retained in **Figure 2A** and in the interpretation of dietary interventions below.

**Figure 2:**
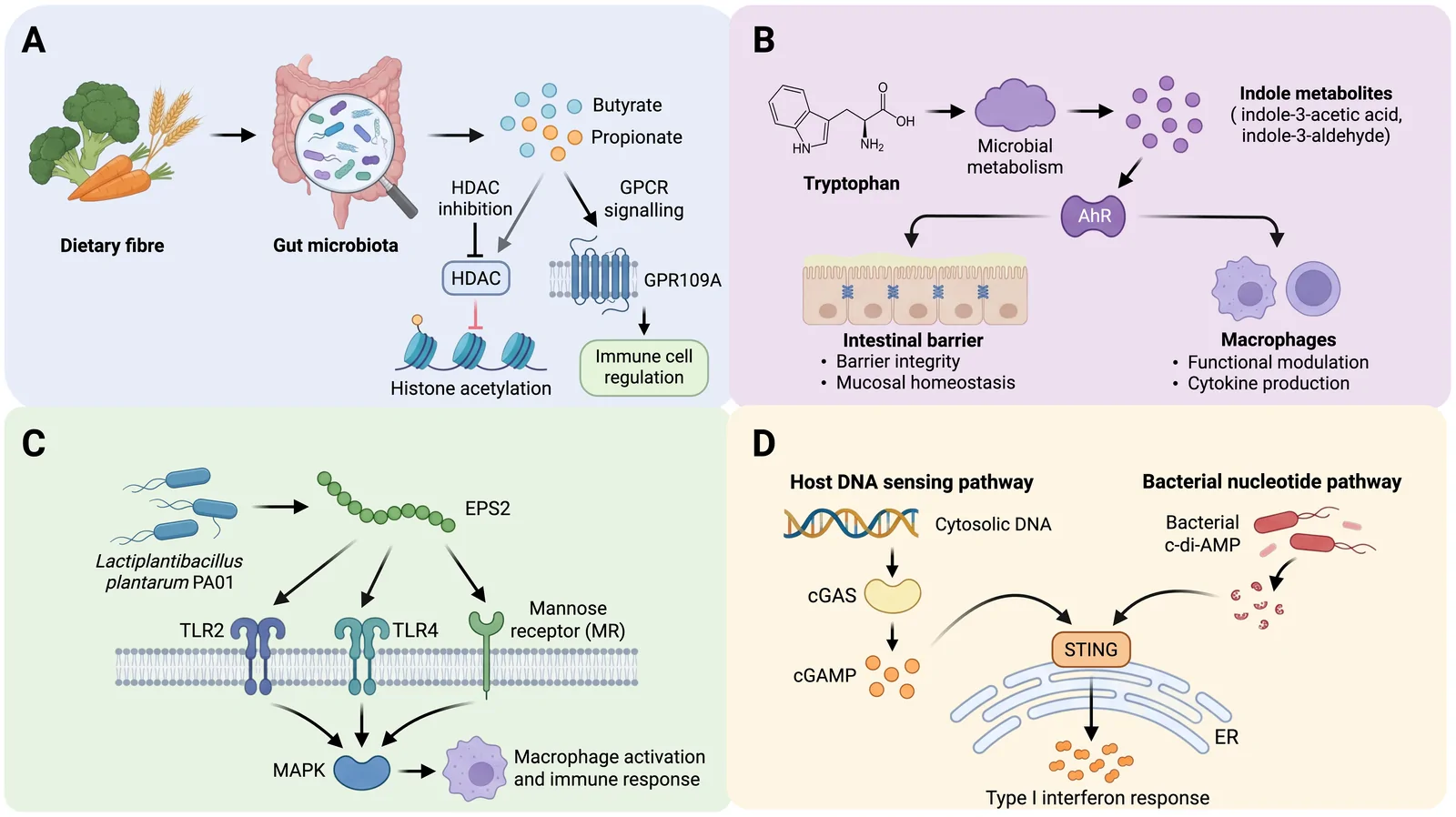
Microbial products and context-dependent regulation of host immune signalling. **(A)** Dietary fibre fermentation by the gut microbiota generates short-chain fatty acids (SCFAs), including butyrate and propionate. Butyrate can inhibit histone deacetylase (HDAC) activity and enhance histone acetylation in a cellular context-dependent manner. SCFAs also regulate immune responses through G-protein-coupled receptors, including GPR109A and FFAR2/FFAR3, with ligand-specific receptor activity. **(B)** Microbial metabolism of dietary tryptophan produces distinct indole derivatives with cell-specific effects. Indole-3-aldehyde (3-IAld) supports intestinal barrier protection through AhR/IL-22-dependent signalling in experimental models of checkpoint-inhibitor-associated colitis. In contrast, selected microbial indoles activate AhR in tumour-associated macrophages, promoting immunosuppressive macrophage programming and reduced antitumour T-cell activity in pancreatic cancer models. **(C)** Exopolysaccharide EPS2 derived from *Lactiplantibacillus plantarum* PA01 modulates macrophage responses through signalling pathways involving Toll-like receptors 2 and 4 (TLR2/TLR4), the mannose receptor (MR) and mitogen-activated protein kinase (MAPK). These responses include enhanced phagocytic activity and inflammatory mediator production in experimental non-tumour models. **(D)** Host cytosolic DNA activates cyclic GMP–AMP synthase (cGAS), generating cyclic GMP–AMP (cGAMP), which subsequently activates stimulator of interferon genes (STING). Bacterial cyclic diadenosine monophosphate (c-di-AMP) can independently activate STING without requiring cGAS-mediated ligand synthesis. In experimental tumour models, microbial STING agonists induce type I interferon signalling in intratumoural monocytes and influence interactions among monocytes, natural killer cells and dendritic cells. Collectively, these pathways illustrate product-, cell- and context-specific immune regulation rather than universally beneficial antitumour effects or established clinical efficacy.

Receptor signaling provides an additional route that is distinct from intracellular HDAC inhibition. In mouse colonic inflammation and cancer models, GPR109A signaling promoted anti-inflammatory properties in macrophages and dendritic cells, supported regulatory T-cell and IL-10-producing T-cell differentiation, and was required for butyrate-associated epithelial IL-18 induction (14). These findings give a defined example of how a metabolite receptor can influence both barrier function and immune differentiation. They do not establish that increasing regulatory cells is beneficial in every established tumor: suppression of inflammation during tumor initiation and suppression of antitumor effector cells during therapy are different endpoints. Receptor abundance, transporter function and the responding cell population must therefore be considered alongside the concentration of the ligand.

Checkpoint blockade makes this contextual distinction particularly clear. Zhu et al. reported that butyrate altered chromatin and T-cell receptor-associated signaling in CD8 T cells and improved anti-PD-1 activity in experimental tumor systems (15). In contrast, Coutzac et al. linked higher circulating butyrate and propionate to poorer outcomes with CTLA-4 blockade and showed in mouse tumor models that butyrate could attenuate treatment-induced immune activation (16). These findings should be presented together rather than combined into a single claim that SCFAs enhance immunotherapy. They involve different therapeutic targets, exposure compartments and immune processes, and neither establishes a universal clinical rule. They instead identify treatment context as a variable that must be prespecified when evaluating a metabolite intervention.

Physiological exposure is a further constraint. Measurements from human intestinal contents and portal, hepatic and peripheral blood show that high colonic SCFA abundance is not equivalent to systemic exposure (17). In a stable-isotope study of 12 healthy participants, the systemic availability of colon-delivered acetate, propionate and butyrate was approximately 36%, 9% and 2%, respectively (18). These values describe that experimental delivery protocol, not a universal bioavailability estimate for all diets or formulations. They nonetheless demonstrate extensive metabolism before peripheral tissues are exposed. A sustained millimolar cell-culture treatment therefore cannot be justified simply by noting that SCFAs are abundant in the colon. Cancer studies should compare the measured concentration, exposure duration, tissue compartment and transporter expression with those used to establish the mechanism; where tumor exposure is unmeasured, physiological relevance remains unresolved.

#### 1.1.2 Tryptophan derivatives: ligand identity and the responding cell

Microbial tryptophan metabolism generates chemically distinct indole derivatives rather than one interchangeable class of AhR agonists. AhR is a ligand-responsive transcription factor whose output depends on the ligand, the responding cell and local cofactors. Zelante et al. linked microbial tryptophan catabolites to AhR-dependent IL-22 activity and mucosal protection in intestinal experimental systems (19). This is useful mechanistic evidence for barrier regulation, but it is not a tumor-efficacy experiment. Similarly, induction of CYP enzymes is a readout of xenobiotic signaling, not proof of immune-mediated cancer control. Changes in CYP activity could alter local drug or hormone handling; demonstration of a therapeutic consequence would additionally require pharmacological exposure and outcome measurements (2). **Figure 2B** therefore depicts receptor engagement and immune regulation without assigning a uniform antitumor direction.

Source attribution is especially important for kynurenine. Opitz et al. identified a tumor-derived tryptophan catabolic route in which host tryptophan 2,3-dioxygenase generated an AhR ligand capable of promoting tumor-cell survival and immune suppression (20). This establishes a host metabolic mechanism, not evidence that the measured kynurenine originated in bacteria. A relationship between kynurenine and microbial community composition may reflect microbial effects on substrate availability or host inflammation without making kynurenine a directly microbial product. Accordingly, kynurenine is retained here as a comparator that illustrates host–microbe ambiguity, and is not classified as a postbiotic. Isotope tracing, producer-specific perturbation and matched host enzyme measurements are needed where a study seeks to assign relative contributions.

A cancer-specific counterpoint to intestinal protection comes from pancreatic ductal adenocarcinoma (PDAC). Hezaveh et al. found that microbial conversion of dietary tryptophan to indoles supported AhR activity in tumor-associated macrophages. Myeloid Ahr deletion or pharmacological inhibition reduced tumor growth and improved checkpoint-blockade activity in mice; dietary manipulations and indole replacement further supported the proposed pathway (21). Human PDAC data associated AhR expression with a suppressive macrophage state and adverse outcomes, but did not constitute a clinical trial of AhR inhibition. The strength of this work lies in its cell-specific perturbations and metabolic tests. Its boundary is that the relevant ligand mixture, dose and macrophage program may differ across tumors. This suppressive pathway is shown separately from intestinal AhR-mediated barrier protection in **Figure 3C**.

**Figure 3:**
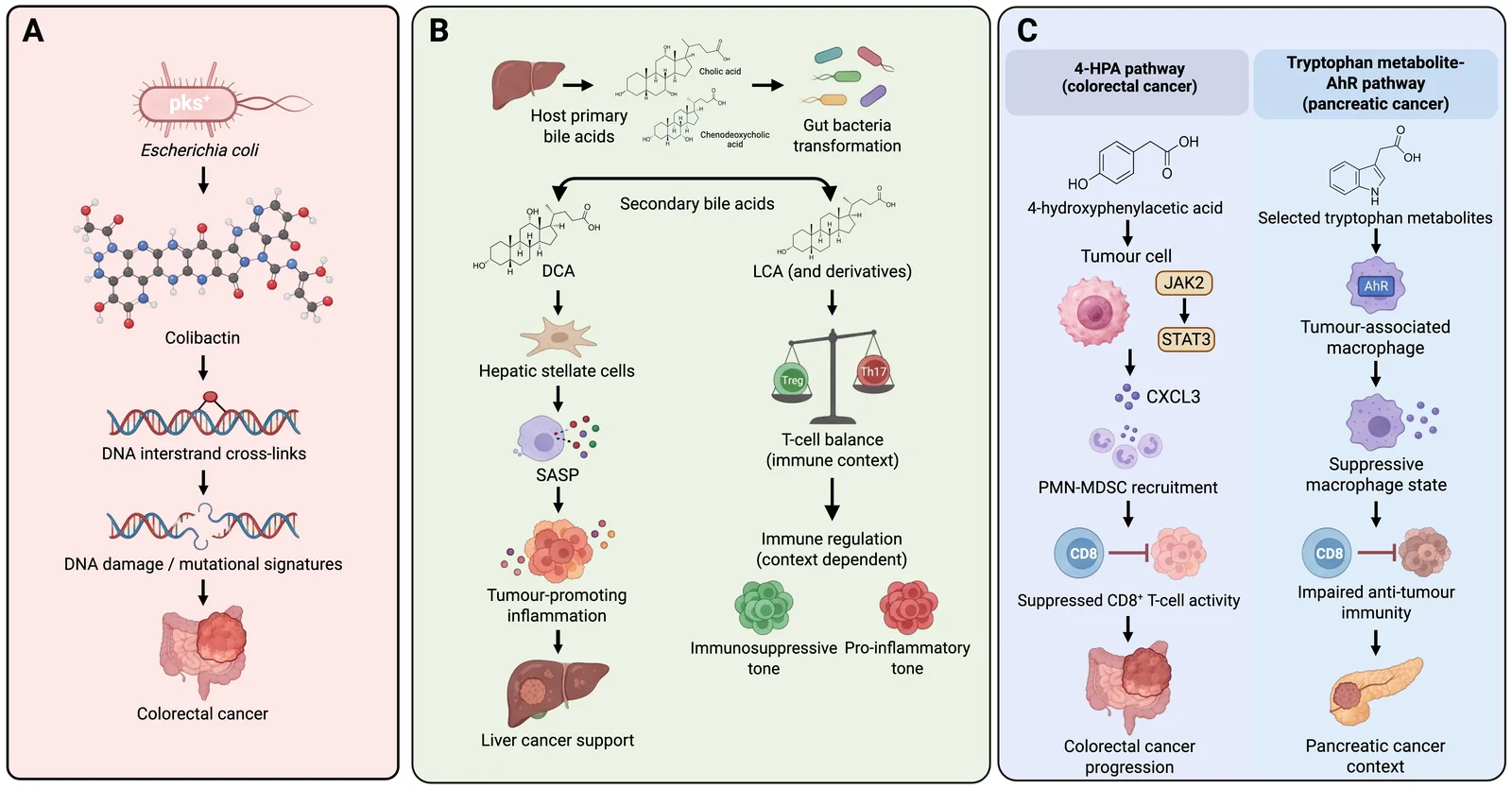
Genotoxic and immunoregulatory pathways mediated by microbial products in cancer. **(A)** Colibactin produced by pks-positive Escherichia coli induces DNA interstrand cross-links and subsequent DNA damage. Colibactin-associated mutational signatures have been identified in experimentally exposed intestinal organoids and human colorectal cancer genomes, supporting a potential contribution to colorectal carcinogenesis. These signatures reflect previous genotoxic exposure rather than necessarily indicating ongoing bacterial activity. **(B)** Host-derived primary bile acids, including cholic acid and chenodeoxycholic acid, undergo microbial transformation in the intestine to generate secondary bile acids, including deoxycholic acid (DCA), lithocholic acid (LCA) and related derivatives. In experimental liver cancer models, DCA promotes a senescence-associated secretory phenotype (SASP) in hepatic stellate cells, contributing to tumour-promoting inflammation. Separately, specific LCA derivatives modulate the differentiation of regulatory T cells (Tregs) and T helper 17 (Th17) cells, influencing immune regulation in a metabolite- and context-dependent manner. These immunoregulatory effects do not establish a uniform tumour-promoting or tumour-suppressive role for secondary bile acids. **(C)** Two distinct microbial metabolite-associated immunosuppressive pathways are illustrated. In colorectal cancer models, 4-hydroxyphenylacetic acid (4-HPA) activates tumour-cell JAK2/STAT3 signalling and increases CXCL3 expression, promoting the recruitment of polymorphonuclear myeloid-derived suppressor cells (PMN-MDSCs), suppression of CD8^+^ T-cell activity and tumour progression. In pancreatic cancer models, selected microbial tryptophan-derived metabolites activate the aryl hydrocarbon receptor (AhR) in tumour-associated macrophages, favouring an immunosuppressive macrophage state and impaired antitumour immunity. These pathways represent distinct, experimentally supported mechanisms, and their relevance to human cancer progression and therapeutic intervention requires further validation. Abbreviations: DCA, deoxycholic acid; LCA, lithocholic acid; SASP, senescence-associated secretory phenotype; Treg, regulatory T cell; Th17, T helper 17 cell; 4-HPA, 4-hydroxyphenylacetic acid; JAK2, Janus kinase 2; STAT3, signal transducer and activator of transcription 3; CXCL3, C-X-C motif chemokine ligand 3; PMN-MDSC, polymorphonuclear myeloid-derived suppressor cell; AhR, aryl hydrocarbon receptor.

Indole-3-carboxaldehyde, also termed indole-3-aldehyde or 3-IAld, provides a more treatment-specific example. Renga et al. tested it in mouse models of checkpoint-inhibitor-associated colitis and used host pathway-deficient animals to implicate AhR/IL-22-dependent barrier protection. The intervention reduced intestinal injury without impairing checkpoint-inhibitor antitumor activity in the tumor models examined (22). This distinguishes control of treatment toxicity from direct tumor killing. It also avoids assuming that every anti-inflammatory effect weakens antitumor immunity. However, preservation of tumor control in those mice cannot establish efficacy or safety in patients receiving different checkpoint regimens. The related metabolite indole-3-acetic acid (3-IAA) is discussed in Section 3.2 because its reported chemotherapy interaction involves oxidative chemistry and autophagy, rather than requiring a general AhR explanation.

#### 1.1.3 Defined microbial products and innate immune signaling

Innate immune effects should be anchored to an identified microbial product and a measured response. For the exopolysaccharide EPS2 purified from Lactiplantibacillus plantarum PA01, Wang et al. reported increased phagocytosis and cytokine or nitric-oxide responses in RAW264.7 macrophages, with receptor and pathway experiments implicating TLR2, TLR4, the mannose receptor and MAPK signaling (23). The same study examined immune restoration in cyclophosphamide-treated mice. These are *in vitro* and immunosuppressed-animal observations; they do not establish tumor regression or efficacy of a different bacterial polysaccharide. Polymer composition, molecular size, purity and potential contamination by other microbial agonists are relevant to interpretation. **Figure 2C** is consequently restricted to the PA01 preparation and its reported immune pathway, avoiding attribution of other strains or NF-κB mechanisms to this single reference.

The cGAS–STING pathway requires an additional distinction between host sensing and direct microbial ligands. Host cGAS detects cytosolic DNA and synthesizes cyclic GMP–AMP, which activates STING and downstream interferon signaling (24). By contrast, bacteria can produce cyclic dinucleotides such as cyclic di-AMP; identification of secreted c-di-AMP from Listeria monocytogenes established that a bacterial second messenger can trigger a host innate response (25). That infection study is mechanistic context, not evidence of cancer treatment. The two routes must not be drawn as if bacterial c-di-AMP first requires cGAS to generate a ligand. **Figure 2D** therefore shows a host DNA–cGAS–cGAMP route and a separate bacterial c-di-AMP route converging on STING.

Direct cancer-relevant support comes from Lam et al., who linked microbiota-derived STING agonists to type I interferon production by intratumoral monocytes in mouse tumor models (26). The resulting changes involved natural killer-cell and dendritic-cell interactions and the balance between immunostimulatory monocytes and tumor-supporting macrophages. Microbiota manipulation and administration of the microbial agonist supported the proposed mechanism, while human melanoma-associated immune patterns and transfer experiments provided translational context. This is stronger evidence than a general statement that STING enhances antigen presentation. It remains a model-dependent pathway, however, and does not establish that all indoles, SCFAs or microbial peptides activate STING. A microbial product, its host sensor and the resulting immune-cell interaction must be identified separately.

Engineering studies further support this distinction. SYNB1891 was designed from E. coli Nissle to generate a STING agonist in a tumor-relevant environment and stimulate phagocytic antigen-presenting cells; preclinical studies measured antitumor responses and incorporated containment features (27). A subsequent phase I study detected pathway engagement in treated human tumors, which moves this specific platform beyond a purely conceptual proposal (28). These results do not validate endogenous microbial metabolite signatures as predictive biomarkers, nor prove comparative clinical benefit. They show how a defined innate agonist can be connected to delivery, pharmacodynamic readouts and early clinical evaluation. The clinical findings and remaining safety requirements are considered in Section 5.2. Together, these examples place innate immunity within a concrete product–sensor–cell framework rather than treating broad immune activation as sufficient evidence of therapeutic value.

### 1.2 Genotoxic and tumor-promoting microbial pathways

#### 1.2.1 Colibactin and the interaction between fiber and genotoxicity

Colibactin produced by pks-positive E. coli is a useful benchmark for stronger causal attribution. Its chemistry supports DNA alkylation and interstrand cross-link formation, with subsequent lesions arising through adduct instability or repair (5). Work using replication systems further implicates the Fanconi anemia pathway in processing these cross-links and shows how lesion bypass can introduce mutations (29). These mechanisms should be distinguished from a nonspecific claim that all bacterial exposure causes DNA breaks. The relevant variables include possession and activity of the biosynthetic machinery, contact with susceptible host cells, and the capacity of those cells to repair the resulting lesions. **Figure 3A** therefore starts with a defined genotoxic bacterial phenotype and follows the DNA lesion rather than labelling colibactin as a broadly acting postbiotic.

Independent experimental approaches connect these molecular lesions to human cancer genomes. Pleguezuelos-Manzano et al. exposed human intestinal organoids to genotoxic pks-positive E. coli and identified mutational patterns also present in colorectal cancers (30). Dziubańska-Kusibab et al. mapped sequence preferences of colibactin-associated DNA damage and related them to mutations in human tumor genomes (31). The convergence of experimental exposure, chemical mechanism and human genomic patterns is more persuasive than a single stool-association study. Nevertheless, a mutational signature records prior mutagenic activity and does not establish the current abundance or activity of bacteria in a particular patient. It also does not by itself validate a screening assay or show that eliminating the organism after tumor formation will improve treatment outcomes.

Dietary fiber is not a contradiction to this mechanism, but its substrate effects require attention. In the ApcMin/+ model studied by Oliero et al., mice underwent DSS exposure and colonization with the pks-positive E. coli strain NC101 before receiving a diet containing 10% inulin or cellulose. Inulin increased bacterial colonization, DNA damage and tumor burden in a manner dependent on colibactin production (32). This differs substantially from the defined butyrate-producing bacterial model in which fiber was protective (13). The studies test different fibers, communities and host conditions, so they cannot be reduced to a binary verdict on fiber. Their common message is that fermentation substrates feed specific microbial activities. Any proposed dietary intervention should therefore identify the expected product and relevant producer ecology, rather than assume that greater fermentation necessarily improves cancer-related outcomes.

#### 1.2.2 Secondary bile acids and distinct forms of immune suppression

Secondary bile acids exemplify host–microbial co-metabolism. Host primary bile acids enter the intestine, where bacterial enzymatic pathways transform them into products including deoxycholic acid (DCA) and lithocholic acid (LCA). Reconstruction of the bile acid dehydroxylation pathway provides direct biochemical and genetic evidence for this microbial conversion (33). A measured bile acid pool nevertheless reflects host synthesis, microbial transformations, intestinal absorption and enterohepatic circulation. It is therefore inappropriate to infer a specific microbial reaction solely from total bile acid abundance. Similarly, DCA, LCA and their derivatives differ chemically and should not be assigned a shared receptor response or a universal pro-tumor role. **Figure 3B** presents the source relationship and divergent biological routes at the level supported by the examples below.

In an obesity-associated liver cancer model, Yoshimoto et al. linked increased DCA to a senescence-associated secretory program in hepatic stellate cells and to inflammatory support for tumor development after carcinogen exposure (34). The causal support came from interventions affecting microbial production and the implicated host-cell state; analogous human tissue findings were supportive but observational. In another liver tumor setting, Ma et al. showed that microbial primary-to-secondary bile acid conversion altered endothelial CXCL16 expression and hepatic CXCR6-positive natural killer T-cell accumulation, thereby modifying antitumor surveillance (35). These are distinct stromal and immune mechanisms, not replications of one identical pathway. Both support attention to the enterohepatic route, while leaving disease-specific human intervention effects to be established.

Related bile acid derivatives can also favor immune regulation rather than the inflammatory effect described above. Hang et al. showed that 3-oxoLCA inhibited Th17 differentiation through RORγt-related activity, whereas isoalloLCA promoted regulatory T-cell differentiation through a mitochondrial reactive-oxygen-species-associated mechanism (36). These intestinal immune experiments clarify structure-specific activity but do not establish cancer benefit or harm. In an established tumor, increased regulatory-cell activity could be interpreted differently from improved control of intestinal inflammation. The apparent conflict therefore cannot be resolved by ranking an entire bile acid class as beneficial or detrimental. It requires specifying the derivative, dose, responding cell and disease stage, and directly testing the cancer endpoint of interest.

The microbial aromatic metabolite 4-hydroxybenzeneacetic acid, also called 4-hydroxyphenylacetic acid or 4-HPA, illustrates a more explicitly defined suppressive route. Liao et al. reported that 4-HPA activated tumor-cell JAK2/STAT3 signaling and increased CXCL3, promoting recruitment of polymorphonuclear myeloid-derived suppressor cells (PMN-MDSCs) and impairment of CD8 T-cell activity in colorectal cancer models (37). Pathway perturbation supported the proposed mechanism, whereas elevated 4-HPA in advanced human colorectal tumors remained an association. This is a single mechanistic program requiring independent replication. **Figure 3C** separates it from macrophage AhR signaling: evidence for PMN-MDSC recruitment does not demonstrate that 4-HPA directly expands regulatory T cells. **Table 1** summarizes the source, model, direction of effect and boundary of each representative pathway.

**Table 1:** Representative microbial products and the boundaries of their cancer-related evidence.

Source and compartment	Product and cancer context	Mechanism and reported direction	Model and evidence type	Interpretation and therapeutic relevance
Gut fermentation; colonic exposure	Butyrate; colorectal cancer	Metabolic utilization versus HDAC inhibition; protection in a defined fiber–producer model (12, 13)	Cells and gnotobiotic mice; experimental causality	Depends on producer, host metabolism and dose; not a universal fiber effect.
Gut-derived; stool or circulating SCFAs	Butyrate/propionate; checkpoint therapy	Favorable anti-PD-1 findings contrast with attenuation of anti-CTLA-4 responses (15, 16, 51)	Mouse intervention; observational and prospective human cohorts	Treatment and compartment differ; no validated universal SCFA threshold.
Microbial tryptophan conversion; gut–tumor axis	Selected indoles; PDAC	Macrophage AhR activity and suppressive immune state (21)	Mouse producer/diet and host-pathway perturbations; human association	Ligand and cell specificity prevent generalization to all AhR ligands.
Microbial tryptophan derivative; intestinal exposure	3-IAld; checkpoint-associated colitis	AhR/IL-22-linked barrier protection with maintained tumor control in tested models (22)	Mouse colitis and tumor experiments	Toxicity protection is not equivalent to direct tumor killing or human benefit.
L. plantarum PA01 preparation	EPS2; immune restoration, not a cancer efficacy model	TLR2/TLR4/mannose-receptor and MAPK-associated macrophage responses (23)	RAW264.7 cells and cyclophosphamide-treated mice	Preparation-specific microbial polymer evidence; not evidence for other strains.
Microbiota-derived bacterial second messenger	c-di-AMP/STING agonists; experimental tumors	STING-dependent type I interferon and innate-cell interactions (26)	Mouse tumor interventions; translational human immune associations	Direct bacterial STING agonism differs from host DNA sensing by cGAS.
pks-positive E. coli; epithelial contact	Colibactin; colorectal cancer	DNA cross-links and mutational signatures; genotoxic direction (5, 29–31)	Biochemistry, cells, organoids and human cancer genomes	Convergent mechanistic support; signatures do not measure current microbial activity.
Host primary bile acids transformed by gut bacteria	DCA/LCA and derivatives; liver/intestinal settings	Senescence-associated tumor promotion or molecule-specific immune effects (33–36)	Mouse cancer and non-cancer immune models; human associations	Host–microbial co-metabolism; individual derivatives cannot share one role label.
Microbial aromatic metabolism; experimental tumor exposure	4-HPA; colorectal cancer	Tumor JAK2–STAT3/CXCL3 and PMN-MDSC recruitment; immune suppression (37)	Preclinical causal experiments with human correlative support	Distinct from regulatory T-cell or macrophage AhR pathways; clinical intervention unvalidated.
B. pseudolongum; gut-derived metabolite	Inosine; checkpoint-treated tumors	A2A-receptor-dependent T-cell support under costimulation (52)	Mouse tumor models; experimental mechanism	Costimulation-dependent; no validated human supplementation strategy.

Directions refer to the specified molecule, exposure, model and endpoint, not an intrinsic property of a metabolite class. Human association is not labeled clinically validated. AhR, aryl hydrocarbon receptor; c-di-AMP, cyclic diadenosine monophosphate; DCA, deoxycholic acid; EPS, exopolysaccharide; HDAC, histone deacetylase; LCA, lithocholic acid; PDAC, pancreatic ductal adenocarcinoma; PMN-MDSC, polymorphonuclear myeloid-derived suppressor cell; SCFA, short-chain fatty acid; 3-IAld, indole-3-aldehyde; 4-HPA, 4-hydroxyphenylacetic acid.

### 1.3 Microbial metabolism and anticancer treatment

#### 1.3.1 Direct drug modification and the limits of clinical inference

Microbial drug metabolism can be resolved at the level of a specific enzyme and chemical product. Geller et al. showed that bacterial cytidine deaminase converts gemcitabine to its inactive deaminated metabolite, 2′,2′-difluorodeoxyuridine (dFdU), and connected this activity to resistance in experimental tumor systems (38). E. coli provides an experimentally tractable example, while the long enzyme isoform is distributed across multiple bacteria, including members of the Gammaproteobacteria. The functional trait should not be assigned indiscriminately to an entire taxonomic class. The causal chain in **Figure 4A** is therefore drug conversion, reduced active exposure and reduced tumor-cell killing, rather than bacterial detection alone. Enzyme activity, bacterial localization and drug concentration are all relevant to whether that chain operates in a patient.

**Figure 4:**
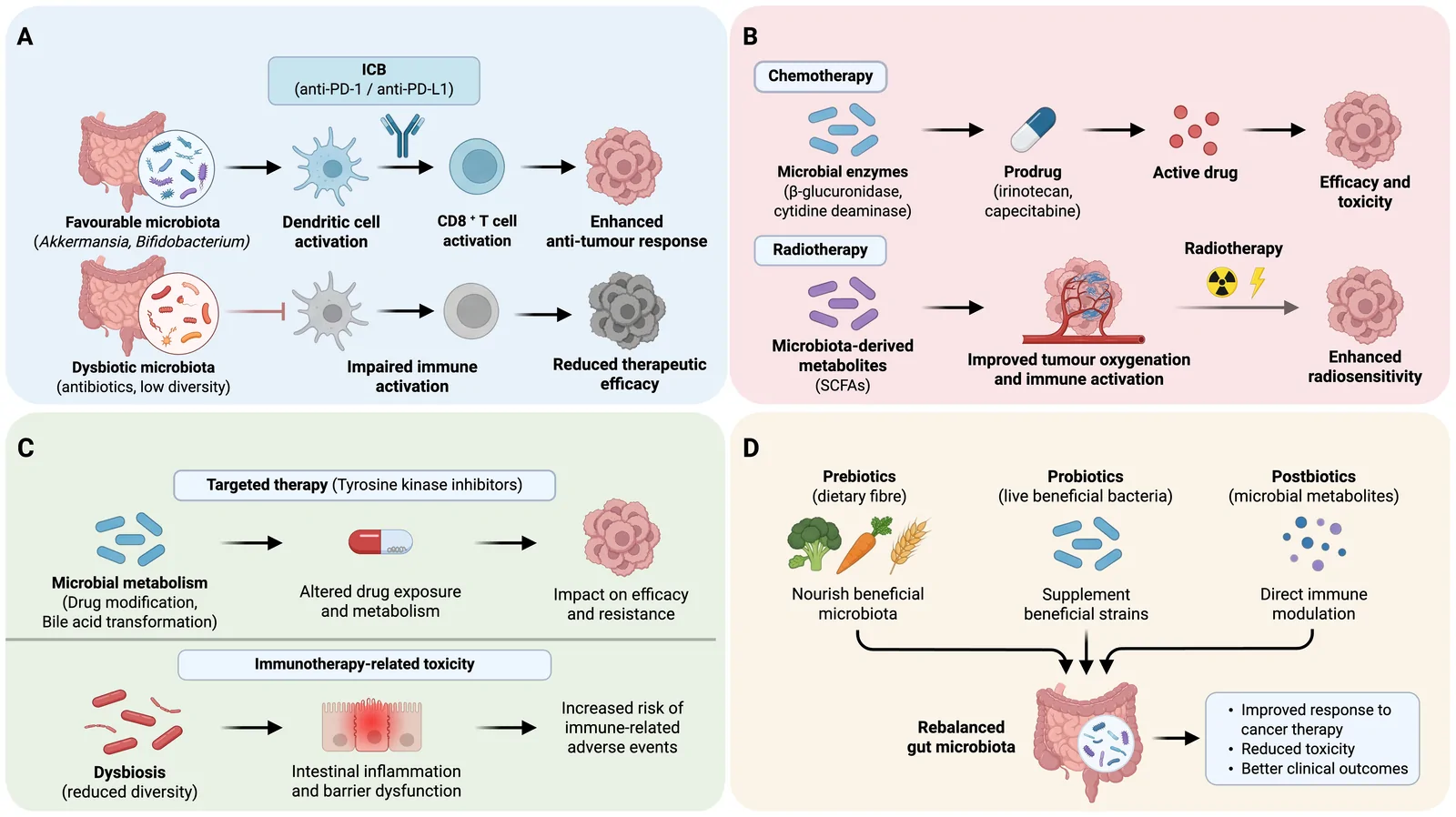
Microbiota-associated modulation of cancer treatment responses and potential therapeutic interventions. **(A)** Gut microbial composition may influence immune checkpoint blockade (ICB) through changes in dendritic cell activation, CD8^+^ T-cell responses and antitumour immunity. Selected microbial communities have been associated with favourable treatment responses, whereas microbial disruption may impair immune activation in specific experimental settings. These associations are context-dependent and do not establish universally beneficial or detrimental microbial profiles. **(B)** Microbial enzymes can modify anticancer drug metabolism, potentially affecting drug activity and toxicity. The consequences depend on the specific drug, enzyme and anatomical compartment. In particular, bacterial β-glucuronidase can reactivate the irinotecan metabolite SN-38 in the intestine, contributing to gastrointestinal toxicity rather than representing the initial activation of irinotecan. The conversion of capecitabine to active fluoropyrimidine metabolites involves host enzymatic pathways and should not be attributed exclusively to microbial enzymes. The radiotherapy branch illustrates experimental associations between microbial metabolism, immune regulation and treatment response. Improved tumour oxygenation is depicted as a potential mechanism rather than an established consequence of the dietary interventions reviewed. **(C)** Microbial metabolism may influence drug exposure and treatment response, while intestinal dysbiosis and barrier dysfunction may contribute to immune-related toxicity. The relationships illustrated for targeted therapy and immunotherapy-associated adverse events are conceptual and require treatment-specific validation. **(D)** Dietary substrates, live microbial preparations and microbial-derived products represent distinct investigational approaches for modifying the intestinal microbiota or host responses. Purified microbial metabolites, although illustrated within the postbiotics category, are distinct from postbiotic preparations under the ISAPP consensus definition. Their potential therapeutic applications require product-specific evaluation of efficacy, toxicity and clinical relevance. Overall, the schematic illustrates possible microbiota–therapy interactions rather than uniformly established causal mechanisms or clinically validated interventions. Abbreviations: ICB, immune checkpoint blockade; PD-1, programmed cell death protein 1; PD-L1, programmed death-ligand 1; SCFAs, short-chain fatty acids; SN-38, active irinotecan metabolite; SN-38G, SN-38 glucuronide.

Human pancreatic tissue findings illustrate why a compelling reaction does not settle its prevalence or clinical importance. May et al. analyzed frozen samples from 26 patients with resected PDAC, including nine long-term and nine short-term survivors after gemcitabine-based neoadjuvant therapy and eight untreated controls; they also examined FFPE samples from five patients (39). Bacterial biomass was low, and Gammaproteobacteria abundance did not correlate with chemotherapy outcomes. The study was small, relied on resection specimens and assessed taxa rather than direct intratumoral drug conversion, so it cannot exclude the enzymatic mechanism. Conversely, the experimental mechanism cannot override the negative human result. A prospective study should measure bacterial enzyme activity and drug/metabolite ratios in appropriately controlled samples alongside treatment exposure and outcomes. **Table 2** explicitly preserves this discordance.

**Table 2:** Treatment interactions and translational stage of representative interventions.

Interaction or approach	Mechanism and direction	Evidence and developmental stage	Discordance or principal limitation	Therapeutic interpretation
Bacterial cytidine deaminase/gemcitabine	Conversion to inactive dFdU; resistance in experimental systems (38)	Biochemical/cell/mouse mechanism; human tissue analyses	A 26-patient human PDAC study did not substantiate the anticipated intratumoral bacterial relationship (39)	Not a validated microbiome-based indication for antibiotics.
Bacterial β-glucuronidase/irinotecan	Gut reactivation of SN-38G to SN-38; inhibitor reduces intestinal toxicity (40)	Preclinical pharmacology	Host activation/glucuronidation must be distinguished from bacterial reactivation	Toxicity mechanism; not direct proof of improved human tumor response.
L. plantarum supernatant/SMCT1	Restored SMCT1 function and butyrate responsiveness (41)	5-FU-resistant HCT116 cells; *in vitro* only	Mixed supernatant; no demonstrated patient efficacy	Mechanistic candidate requiring product definition and *in vivo* validation.
3-IAA/chemotherapy	Oxidative pathway, ROS accumulation and reduced autophagy (42)	PDAC mouse mechanism; two independent human cohort associations	Human correlation does not establish supplementation efficacy	Preclinical sensitizer with clinical associative support.
Pentadecanoic acid/oxaliplatin	FUBP1-related glycolytic changes and sensitization (43)	Preclinical gastric cancer	Tumor exposure and controlled clinical benefit unresolved	Not an established oncology adjunct.
Diet and selected fiber formulations	Changes in microbial metabolism and radiotherapy response (54)	Mouse bladder tumor models; separate observational dietary evidence (53)	Formulation dependence; adverse inulin–colibactin result in a different model (32)	Cancer-treatment efficacy requires product-specific trials.
FMT with anti-PD-1 re-treatment	Community transfer with clinical benefit in 6/15 or objective responses in 3/10 (55, 56)	Early uncontrolled clinical studies in refractory melanoma	Different endpoints; small cohorts; no attribution to one metabolite	Early clinical, not established metabolite therapy.
Targeted phage–nanoparticle system	F. nucleatum targeting with irinotecan delivery (57)	Mouse colorectal tumor models; preclinical	Host range, resistance, delivery and mediator attribution	Selective microbial targeting remains investigational.
Engineered arginine or nanobody delivery	Local metabolic modification or protein payload release (58–60)	Mouse tumor models; preclinical	Nanobodies are not metabolites; construct-specific safety	Platforms cannot be assigned a shared clinical stage.
SYNB1891 STING-agonist delivery	Intratumoral bacterial production and pathway engagement (27, 28)	Phase I: 24 monotherapy and 8 combination participants	Cytokine release including a dose-limiting event; efficacy not established	Early clinical target engagement for this specific construct.
Intravenous sodium butyrate	Systemic defined-metabolite exposure (62)	Historical human pharmacology; nine acute leukemia patients	39–59 μM plasma levels; approximately six-minute half-life; no clear efficacy	Human dosing experience does not establish an effective anticancer regimen.
Indole-3-propionic-acid microcapsules	Protected intestinal delivery with prebiotic materials (63)	Mouse colitis; preclinical non-cancer formulation	No cancer efficacy test in the cited study	Enabling delivery evidence only.

Stages describe the cited application, not all uses of the intervention, and do not imply marketing approval. None of these examples establishes a routine metabolite-directed anticancer treatment. dFdU, 2′,2′-difluorodeoxyuridine; FMT, fecal microbiota transplantation; FUBP1, far upstream element-binding protein 1; ROS, reactive oxygen species; SMCT1, sodium-coupled monocarboxylate transporter 1; SN-38, active irinotecan metabolite; SN-38G, SN-38 glucuronide; 3-IAA, indole-3-acetic acid.

Irinotecan illustrates a different anatomical and pharmacological process. Host enzymes generate the active metabolite SN-38 and glucuronidate it to SN-38G; after intestinal delivery, bacterial β-glucuronidases can regenerate SN-38 and increase epithelial exposure. Wallace et al. developed selective bacterial enzyme inhibitors that reduced irinotecan-associated intestinal toxicity in mice (40). **Figure 4B** separates host activation and conjugation from microbial deconjugation so that intestinal toxicity is not confused with tumor-local drug inactivation. The principal experimental endpoint is protection from gastrointestinal injury, not proof of improved survival. Translation requires showing that a selective inhibitor reduces clinically relevant toxicity without compromising systemic anticancer exposure. This example also explains why community depletion and enzyme-specific inhibition are not interchangeable interventions: they affect different biological functions and have different consequences for other microbial activities.

#### 1.3.2 Chemosensitizing metabolites and treatment-specific mechanisms

The butyrate–SMCT1 interaction concerns metabolite uptake rather than direct modification of an anticancer drug. Kim et al. studied HCT116 cells selected for 5-fluorouracil resistance and found reduced expression and function of the sodium-coupled monocarboxylate transporter SMCT1, accompanied by diminished sensitivity to butyrate. A cell-free L. plantarum culture supernatant restored transporter function and butyrate-associated antiproliferative and apoptotic responses (41). The evidence is an *in vitro* mechanism in a resistant colorectal cell line. The preparation is a mixture, so the experiment does not identify one purified bacterial molecule as the transporter-restoring agent. It also does not establish clinical reversal of 5-fluorouracil resistance. **Figure 4C** identifies butyrate uptake through SMCT1 and the model restriction explicitly; patient relevance would require transporter measurements, defined active components and tumor exposure data.

For 3-IAA, Tintelnot et al. combined human PDAC metabolomic observations with gnotobiotic mouse experiments, dietary manipulation and metabolite administration (42). Their mechanistic work implicated neutrophil myeloperoxidase-dependent oxidation of 3-IAA, accumulation of reactive oxygen species and suppression of tumor-cell autophagy during chemotherapy. Associations between 3-IAA and chemotherapy efficacy were observed in two independent human cohorts, which is stronger than a single discovery correlation. However, patients were not randomized to 3-IAA treatment, and the human associations therefore do not prove benefit from supplementation. The study is best interpreted as an experimentally supported therapeutic hypothesis with human relevance. It also shows why generic attribution of all indole effects to AhR is inadequate: the relevant mechanism here depends on oxidative processing and the chemotherapy context.

Pentadecanoic acid links a defined microbial metabolic product to chemotherapy sensitivity in a different disease setting. Xu et al. linked metabolically active Akkermansia muciniphila and its associated pentadecanoic acid production to oxaliplatin sensitivity in gastric cancer models. The proposed mechanism involved antagonism of FUBP1-dependent glycolysis, and the experiments combined microbial manipulation, metabolic profiling and metabolite supplementation (43). This is preclinical evidence and does not establish pentadecanoic acid as a treatment for gastric cancer patients. It also cannot be substituted for the PDAC-specific 3-IAA mechanism or the cell-based SMCT1 finding. These examples are separated in **Figure 4C** because a shared description as chemosensitization conceals differences in chemical target, cancer model, combination treatment and evidential maturity.

### 1.4 From functional profiling to clinically useful biomarkers

#### 1.4.1 Metabolite identity, source attribution and metabolic activity

A functional profiling strategy must distinguish metabolic potential, expression, concentration and flux. A 16S rRNA profile measures taxonomic markers; prediction from reference genomes estimates possible pathway content but cannot establish expression or metabolite production in the sampled patient. Benchmarking by Matchado et al. documents limitations of 16S-based functional prediction (44). Shotgun metagenomics can identify relevant genes, metatranscriptomics can assess transcription, and enzyme assays can test catalytic capacity. Direct metabolomics measures chemical abundance, while isotope tracing and controlled perturbations are needed to resolve production and utilization. The culture-based approach of Han et al. illustrates how producer assignments can be strengthened experimentally (6). These measurements are complementary: none alone demonstrates that an observed concentration is sufficient to alter a host pathway.

Concentration also differs from metabolic activity because it reflects production, transport, consumption and clearance. A low circulating concentration may coexist with substantial local turnover, while a high concentration may reflect impaired host clearance rather than increased microbial synthesis. Targeted assays should therefore confirm compound identity against an authentic standard and quantify the relevant compartment, with attention to sampling time, storage, analytical batch and internal standards. For tumor samples, extraction blanks, handling controls, adjacent tissue and independent detection methods are especially important because bacterial biomass can approach background levels (3, 39). If a biosynthetic gene is detected but the product is not measured, the conclusion should remain one of potential capacity. If a compound is measured without a demonstrated producer, microbial origin should remain a hypothesis.

Exploratory multi-omics reports are useful when their sample sizes and design limits are visible. Liu et al. compared stool microbiota and metabolites in 52 patients with non-small-cell lung cancer and 29 healthy participants and reported stage-associated metabolic differences (45). These cross-sectional results nominate candidate associations; they cannot show that an altered metabolite drives progression or predict an individual patient’s future stage. Luo et al. profiled 47 paired pancreatic cancer and adjacent tissues and found microbiome–metabolome correlations, including changes involving amino acid pathways (46). Paired sampling improves within-patient comparison, but a correlation between Pseudomonas signals and a tissue metabolite does not establish bacterial synthesis of that metabolite. These studies should be interpreted alongside contamination-sensitive or negative pancreatic findings rather than presented as uniformly confirming a tumor-specific microbial function.

#### 1.4.2 Immunotherapy biomarkers: replication, confounding and clinical utility

Several human studies associated gut microbiome composition with checkpoint-inhibitor outcomes in melanoma and epithelial cancers, with transfer experiments supporting a contribution of the microbial community (47–49). These observations justify functional biomarker research but are not direct validation of a circulating metabolite assay. A cross-cohort analysis of five observational melanoma cohorts comprising 165 participants, integrated with 147 previously published samples, found cohort-dependent microbiome associations and limited transferability of specific signatures (50). This is evidence about taxonomic classifiers, not a direct test of every metabolite marker. It nevertheless illustrates why favorable results in one population cannot establish a universal assay. Differences in geography, clinical practice, diet and analytical methods must be considered before defining a responder-associated metabolic state.

A direct metabolite example is the prospective cohort studied by Nomura et al.: pretreatment fecal and plasma SCFAs were measured in 52 patients receiving nivolumab or pembrolizumab, and higher concentrations of selected SCFAs were associated with longer progression-free survival (51). Prospective sampling gives the marker temporal precedence over the outcome, but the study remains observational and includes heterogeneous tumors. Its findings should be read alongside the less favorable systemic SCFA associations during CTLA-4 blockade discussed in Section 1.1 (16). Differences between stool and blood, PD-1 and CTLA-4 inhibition, and the associated immune responses prevent a simple shared threshold. Thus, “SCFA-high” is not a clinically validated category, and a favorable fecal association does not justify increasing systemic butyrate in every patient.

Microbial inosine supplies a distinct mechanistic example. Mager et al. linked Bifidobacterium pseudolongum-derived inosine to improved checkpoint-blockade activity in mouse tumor models, with T-cell adenosine A2A receptor signaling and a requirement for costimulation (52). This explains why the receptor name alone does not determine the direction of immune regulation: the activation context matters. The work supports a model-specific effector mechanism, not a validated human inosine assay or a general recommendation for supplementation. A metabolite can therefore be a plausible causal target while its biomarker performance remains unknown; conversely, an outcome-associated marker may be useful for prediction even if it is not a therapeutic effector. The validation paths for these uses should remain distinct.

Clinical confounding needs to be addressed at study design rather than appended as a generic limitation. Diet affects substrate supply; antibiotics alter producers; proton-pump inhibitors can change gastrointestinal ecology; corticosteroids affect immune activity and may mark treatment complications. Age, sex, BMI, geography, cancer stage, organ function, previous therapy and the checkpoint regimen may influence both exposure and outcome. Spencer et al. combined patient observations and mouse experiments to examine fiber, probiotic use and melanoma immunotherapy, illustrating the importance of recording diet and microbial interventions (53). These findings do not establish a uniform probiotic or fiber prescription. For biomarker development, the practical response is prespecified exposure windows, pretreatment and longitudinal sampling, careful medication records and adjustment for relevant clinical factors, while recognizing residual confounding.

The term predictive should be reserved for a clearly defined use. A marker associated with outcome among treated patients may be prognostic, associated with general health or linked to treatment exposure; proving prediction of differential treatment benefit usually requires a treatment comparison and an interaction analysis. An externally tested model should use a locked assay, prespecified thresholds and a defined outcome, and should report discrimination, calibration and uncertainty rather than an optimized discovery-cohort accuracy alone. Its incremental value over clinical predictors and the consequences of acting on the result must also be examined, consistent with transparent tumor-marker reporting (11). We therefore classify current metabolite signatures according to the evidence for their stated use, rather than assume that biological plausibility or a significant correlation establishes clinical utility.

### 1.5 Translational strategies and their developmental stage

#### 1.5.1 Diet, community interventions and selective microbial targeting

Dietary intervention can change several metabolites simultaneously, making both benefit attribution and standardization difficult. In mice with bladder tumor allografts, Then et al. found that selected fiber combinations improved radiotherapy-associated tumor control and altered intestinal microbial or metabolic profiles, with some formulations also reducing intestinal injury (54). This is cancer-relevant preclinical evidence, but the fiber formulation, background diet and radiation regimen are integral to the result. It should be compared with the inulin–colibactin interaction described in Section 2.1 rather than generalized to all fiber supplementation. Translation would require a specified dietary product, adherence assessment, measurement of candidate mediators and separate efficacy and toxicity endpoints. General nutritional support and an intervention intended to alter antitumor efficacy should not be assigned the same evidentiary status.

Fecal microbiota transplantation (FMT) has progressed further in selected immunotherapy settings. Two early studies combined donor FMT with anti-PD-1 re-treatment in refractory melanoma. Davar et al. reported clinical benefit in 6 of 15 participants, while Baruch et al. observed three objective responses among 10 participants (55, 56). Clinical benefit and objective response are different endpoints and should not be combined into one response rate. These small, uncontrolled studies demonstrate feasibility and treatment-associated biological changes, but cannot isolate the effects of FMT, anti-PD-1 re-exposure or a specific metabolite. FMT in this oncology context is therefore an early clinical strategy rather than an established metabolite therapy. Defined live biotherapeutics and probiotics likewise require strain-, dose- and indication-specific testing; a commercial probiotic cannot be assumed to reproduce the community function transferred by FMT.

As illustrated in **Figure 4D**, prebiotic approaches, probiotics and microbial-derived products represent distinct strategies for modulating the gut microbiota and host responses during cancer treatment. Prebiotic substrates may influence microbial composition and metabolic activity, whereas probiotics introduce selected live microorganisms and microbial-derived products may exert more direct effects on host signalling pathways. Importantly, purified microbial metabolites should not automatically be classified as postbiotics, which are defined as preparations of inanimate microorganisms and/or their components that confer a health benefit. Although these approaches have shown potential to influence treatment responses and therapy-associated toxicity in selected experimental settings, their effects are context dependent, and improvements in clinical outcomes remain insufficiently established. Thus, the interventions depicted in **Figure 4D** should be regarded as investigational strategies rather than universally effective methods of restoring microbial balance or enhancing anticancer therapy.

Selective targeting offers another investigational route. In colorectal tumor models, Zheng et al. used phages directed against Fusobacterium nucleatum together with irinotecan-loaded dextran nanoparticles, improving chemotherapy effects in mice (57). This primary study provides a basis for the phage option shown in **Figure 5**, while not proving that its effect was mediated by one measured microbial metabolite. Phage host range, resistance, ecological consequences and delivery determine whether such a strategy changes the intended function. Small-molecule inhibitors offer a different approach by acting on a defined enzyme, as illustrated by bacterial β-glucuronidase inhibition. Intervention selection should follow the demonstrated mechanism: removal of a producer, inhibition of an enzyme and replacement of a missing molecule require different validation and should not be treated as equivalent methods of “microbiome correction”.

**Figure 5:**
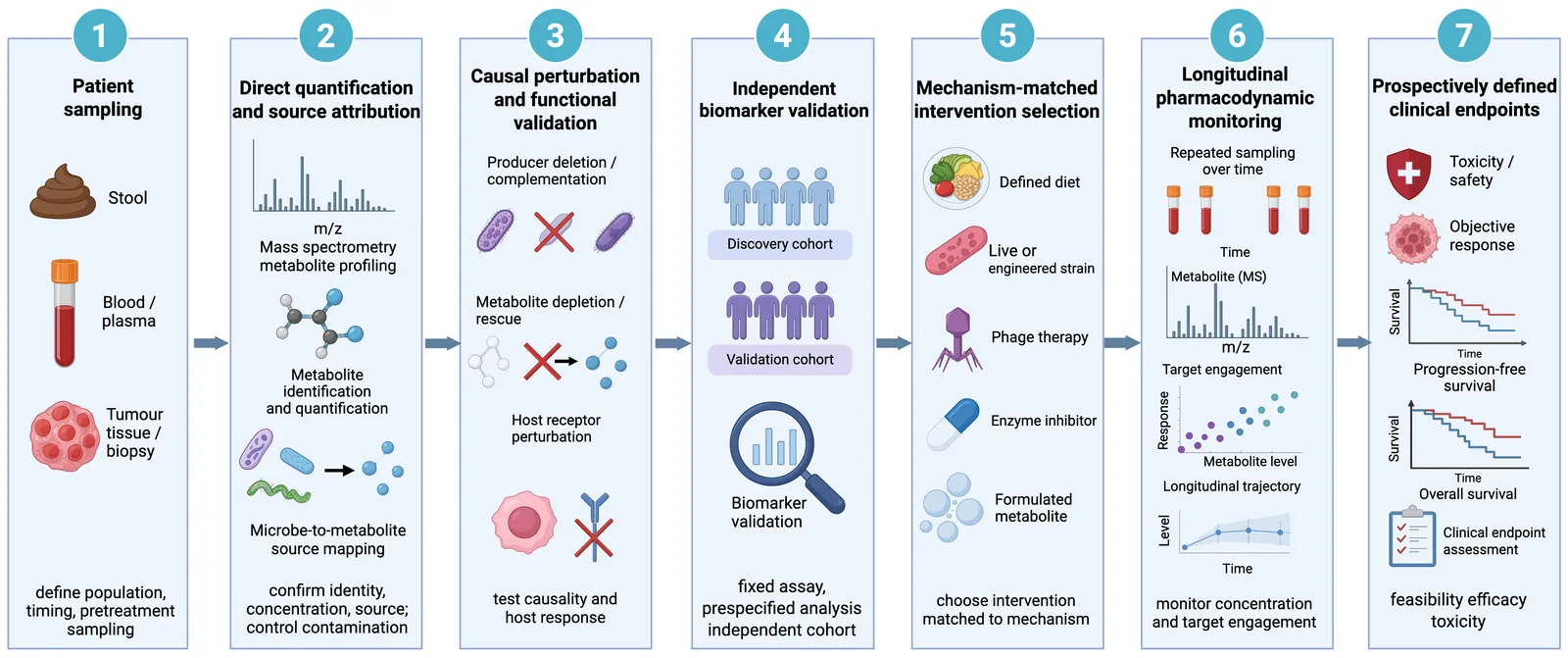
A proposed seven-step framework for translating microbial metabolite research into clinical applications. The framework integrates seven complementary stages: (1) patient sampling, including stool, blood and tumour tissue collection from defined populations; (2) direct metabolite identification and quantification using analytical approaches such as mass spectrometry, together with microbial source attribution; (3) causal perturbation and functional validation through microbial producer deletion or complementation, metabolite depletion and rescue, and host receptor or pathway perturbation; (4) independent biomarker validation using discovery and validation cohorts, fixed analytical assays and prespecified evaluation criteria; (5) selection of mechanism-matched interventions, including defined dietary approaches, live or engineered microbial strains, phage therapy, enzyme inhibitors and formulated metabolites; (6) longitudinal pharmacodynamic monitoring to assess metabolite exposure, changes in host pathway activity and target engagement; and (7) evaluation of prospectively defined clinical endpoints, including safety, toxicity, objective tumour response, progression-free survival and overall survival. Biomarker validation and mechanism-guided intervention development represent complementary translational pathways rather than universally mandatory sequential stages. Changes in metabolite concentrations alone do not establish target engagement, which requires corresponding evidence of host pathway modulation. Confounder control, analytical standardisation, biosafety, manufacturing consistency and regulatory assessment should be considered throughout the translational process. This framework represents the authors' conceptual synthesis and requires prospective validation. All mass spectra, scatterplots, longitudinal trajectories and survival curves are schematic illustrations and do not represent empirical patient data or predict treatment effects.

#### 1.5.2 Engineered bacteria: metabolic delivery, early trials and biosafety

Engineered bacteria can deliver a therapeutic product or modify the local metabolic environment, and these are distinct design objectives. Canale et al. engineered E. coli Nissle to convert tumor-associated ammonia into L-arginine, increasing local arginine availability and supporting T-cell-dependent responses to PD-L1 blockade in mice (58). This is a direct example of local metabolic engineering. By comparison, Gurbatri et al. used a controlled lysis system to deliver checkpoint-blocking nanobodies (59), and Chowdhury et al. delivered an anti-CD47 nanobody with local and systemic antitumor effects in mouse models (60). Nanobodies are protein payloads, not microbial metabolites. These studies are included to explain delivery and control strategies, with their contribution separated from evidence for the activity of an endogenous small molecule.

The clinical stage must be assigned to the specific construct. In the phase I SYNB1891 study, 24 participants received intratumoral monotherapy and eight received combination treatment with atezolizumab. Tumor biopsies showed STING-pathway target engagement; five cytokine-release-syndrome events occurred with monotherapy, including one dose-limiting event, and no SYNB1891-related infections were reported (28). These findings support early clinical feasibility and pharmacodynamic activity, not established efficacy or safety of engineered bacteria as a class. They also distinguish local injection into accessible lesions from systemic delivery to disseminated disease. A review should therefore state that many constructs remain preclinical while selected platforms have entered phase I, rather than categorize the entire field as conceptual or imply that one early trial establishes routine clinical use.

Biosafety requires testing the living chassis as well as the payload. Bacteremia and uncontrolled colonization depend on the organism, route, host immune status and clearance; immunogenicity or cytokine release may occur even when viable bacteria are not recovered from blood. Biodistribution studies should assess tumor localization, off-target organs, persistence and antibiotic susceptibility. Genetic instability can change product output during replication, while horizontal gene transfer can separate a therapeutic or resistance element from its intended host. Reviews of bacterial delivery identify these as central translational constraints (10). Consequently, tumor shrinkage in a mouse experiment is insufficient to establish a usable product. An informative preclinical program must also define how exposure can be monitored and terminated and whether the intended metabolic function remains stable over time.

Containment strategies should be evaluated for their failure modes rather than described as guaranteed safeguards. Auxotrophy or dependence on a non-standard nutrient can restrict growth, and kill-switch circuits can make survival conditional on environmental signals. Mandell et al. demonstrated synthetic nutrient dependence in engineered E. coli, providing an enabling biocontainment approach rather than an oncology efficacy result (61). Escape mutation, environmental complementation and genetic transfer must still be assessed for the actual therapeutic strain. Environmental shedding requires a sampling and clearance plan, while manufacturing must control identity, viable dose, purity, genetic integrity and product potency. Regulatory classification and the associated development package depend on whether the intervention is a live genetically modified organism, a purified molecule or a combination product; they cannot be inferred from the informal term postbiotic. SYNB1891 illustrates integration of these considerations into a defined development program (27).

#### 1.5.3 Defined metabolite administration and formulation

Direct administration has the advantage of a measurable chemical dose, but host clearance can prevent the exposure required by cell experiments. A small clinical pharmacology study treated nine patients with acute leukemia by continuous intravenous sodium butyrate. Plasma concentrations reached 39–59 μM, with a reported post-infusion half-life of approximately six minutes and no clear clinical efficacy (62). This historical study is not a modern efficacy assessment, but it is direct human evidence of a major exposure limitation. It also makes clear why it would be inaccurate to claim that no human metabolite administration has ever been studied. The appropriate conclusion is that such human pharmacological observations and predominantly preclinical combination data do not establish routine anticancer benefit from purified butyrate, indoles or related compounds.

Delivery systems should therefore be judged against a defined target compartment. Oral encapsulation may protect a compound during transit and favor intestinal release, whereas systemic or intratumoral delivery aims at different exposure profiles. Yang et al. developed microcapsules combining indole-3-propionic acid with prebiotic materials and demonstrated protection in mouse colitis (63). This is primary delivery evidence for intestinal inflammation, not a demonstration of cancer efficacy; the terminology used by that study does not change the postbiotic definition adopted here. For oncology, a formulation must show release, stability, target-tissue concentration and the intended host response, followed by combination-specific toxicity and efficacy testing. Physical encapsulation alone cannot establish that a metabolite reaches a distant tumor or preserves a favorable therapeutic window.

Combination studies also need to disentangle tumor sensitization from altered drug exposure or protection of normal tissue. The irinotecan example concerns intestinal drug reactivation; 3-IAA acts within a defined chemotherapy-associated oxidative mechanism; 3-IAld was tested for reduction of checkpoint-associated intestinal injury (22, 40, 42). Each requires a different pharmacodynamic endpoint. A credible dose-finding design would measure both the parent metabolite and relevant transformation products, assess activity at the proposed host target, and record anticancer efficacy alongside organ toxicity. These are research requirements rather than a claim that an effective regimen has been identified. **Table 2** therefore distinguishes enzyme inhibition, metabolite supplementation, community modulation and engineered delivery and records the clinical stage supported by each example.

#### 1.5.4 A seven-step framework for testing translational claims

**Figure 5** is a proposed research framework, not a validated clinical algorithm. Step 1 defines the patient population and sampling plan, including pretreatment blood, stool and tumor material when appropriate. Step 2 confirms metabolite identity and concentration and tests its microbial or host–microbial source. Steps 1 and 2 should specify treatment timing, pre-analytical procedures, reference standards and contamination controls before examining associations. Their purpose is to prevent a predicted pathway or an unverified mass-spectrometry feature from being promoted directly into a clinical target. The framework draws on direct producer mapping and the recognized limits of sequencing-based functional inference (6, 44); the ordered sequence itself is the authors’ synthesis. Step 3 tests causality and function through producer-gene deletion or complementation, metabolite depletion and rescue, host receptor perturbation and relevant tumor or immune readouts. Gnotobiotic butyrate experiments and the 3-IAA chemotherapy study illustrate different implementations of this logic (13, 42). Step 4 separately tests biomarker performance in independent patients using a fixed assay and prespecified analysis. A reproducible biomarker need not itself be causal, but using it to select a treatment requires evidence for that intended use. Conversely, a causal experimental effector may have poor predictive value if its concentration fluctuates or does not capture the relevant tissue exposure. This separation is the framework’s main practical contribution relative to a simple profile–predict–intervene sequence. Step 5 selects an intervention that matches the validated mechanism, such as a defined diet, a live or engineered strain, a phage, an enzyme inhibitor or a formulated metabolite. Step 6 monitors pharmacodynamics longitudinally, distinguishing a change in concentration from engagement of the host target. The SYNB1891 trial illustrates the latter distinction through tumor pathway measurements (28). Step 7 evaluates prospectively defined clinical endpoints, including toxicity, objective response, progression-free survival or overall survival as appropriate to the trial phase. Early studies should prioritize feasibility and target engagement; comparative efficacy requires a suitable controlled design. The illustrative plots in **Figure 5** contain no study data and predict no treatment effect. Confounder control, analytical standardization, biosafety, manufacturing consistency and regulatory assessment apply across all seven steps.

## 2 Conclusion

Microbial products affect cancer through several experimentally distinguishable processes: chemical DNA damage, receptor and innate-immune signaling, alteration of host metabolic state and direct drug conversion. The strongest causal evidence is attached to specific molecules and models, not to a universally favorable microbiome or metabolite profile. Human studies provide important associations, early intervention feasibility and selected pharmacodynamic observations, but the bridge to clinical efficacy remains uneven. Negative pancreatic microbiome findings, opposing SCFA effects across checkpoint settings and substrate-dependent colibactin activity are informative boundaries that should guide experimental design rather than be treated as exceptions to a generally therapeutic narrative.

Three priorities follow from the evidence reviewed here. First, pair spatially and temporally appropriate metabolite measurement with producer and host-target perturbation, so that source and mechanism are tested together. Second, evaluate prespecified metabolite biomarkers in independent, clinically characterized cohorts, explicitly distinguishing prognosis, treatment prediction and target engagement. Third, advance the best-supported interventions through exposure, safety and controlled clinical studies with separate efficacy and toxicity endpoints. These priorities allow microbial metabolism to be assessed as a set of testable biological and therapeutic relationships, while preserving the uncertainty required for responsible translation.
